# Oxalyltransferase, a plant cell‐wall acyltransferase activity, transfers oxalate groups from ascorbate metabolites to carbohydrates

**DOI:** 10.1111/tpj.13984

**Published:** 2018-07-26

**Authors:** Rebecca A. Dewhirst, Stephen C. Fry

**Affiliations:** ^1^ The Edinburgh Cell Wall Group Institute of Molecular Plant Sciences The University of Edinburgh Edinburgh EH9 3BF UK; ^2^ Present address: wildFIRE lab Hatherly Laboratories Prince of Wales Road University of Exeter Exeter UK

**Keywords:** oxalyl‐sugars, vitamin C, cell‐wall modification, oxalic acid, apoplast, cell‐suspension culture, oxalyl‐threonate, spinach, Arabidopsis

## Abstract

In the plant apoplast, ascorbate is oxidised, via dehydroascorbic acid, to *O*‐oxalyl esters [oxalyl‐l‐threonate (OxT) and cyclic oxalyl‐l‐threonate (cOxT)]. We tested whether OxT and cOxT can donate the oxalyl group in transacylation reactions to form oxalyl‐polysaccharides, potentially modifying the cell wall. [*oxalyl*‐^14^C]OxT was incubated with living spinach (*Spinacia oleracea*) and Arabidopsis cell‐suspension cultures in the presence or absence of proposed acceptor substrates (carbohydrates). In addition, [^14^C]OxT and [^14^C]cOxT were incubated *in vitro* with cell‐wall enzyme preparations plus proposed acceptor substrates. Radioactive products were monitored electrophoretically. Oxalyltransferase activity was detected. Living cells incorporated oxalate groups from OxT into cell‐wall polymers via ester bonds. When sugars were added, [^14^C]oxalyl‐sugars were formed, in competition with OxT hydrolysis. Preferred acceptor substrates were carbohydrates possessing primary alcohols e.g. glucose. A model transacylation product, [^14^C]oxalyl‐glucose, was relatively stable *in vivo* (half‐life >24 h), whereas [^14^C]OxT underwent rapid turnover (half‐life ~6 h). Ionically wall‐bound enzymes catalysed similar transacylation reactions *in vitro* with OxT or cOxT as oxalyl donor substrates and any of a range of sugars or hemicelluloses as acceptor substrates. Glucosamine was *O*‐oxalylated, not *N*‐oxalylated. We conclude that plants possess apoplastic acyltransferase (oxalyltransferase) activity that transfers oxalyl groups from ascorbate catabolites to carbohydrates, forming relatively long‐lived *O*‐oxalyl‐carbohydrates. The findings increase the range of known metabolites whose accumulation *in vivo* indicates vitamin C catabolism. Possible signalling roles of the resulting oxalyl‐sugars can now be investigated, as can the potential ability of polysaccharide oxalylation to modify the wall's physical properties.

## Significance Statement

Several major metabolites of vitamin C (oxalyl‐threonates and cyclic oxalyl‐threonate) contain oxalyl ester groups, which we now demonstrate can be transferred onto plant carbohydrates by an oxalyltransferase activity. Such transacylation reactions may represent mechanisms of cell‐wall polymer remodelling (introducing strongly acidic oxalyl side‐chains) as well as generating low‐molecular‐weight oxalyl‐sugars that are potential signalling molecules and could serve as ‘fingerprints’ indicating the presence of apoplastic ascorbate catabolites.

## Introduction

Ascorbate is the most abundant low‐molecular‐weight antioxidant in plants, and some of it is present in the apoplast (the fluid that permeates the cell wall) (Takahama, 1993; Polle *et al*., 1995; Liso *et al*., 2004; Zechmann *et al*., 2011) Within the plant, ascorbate can be oxidised to form dehydroascorbic acid (DHA) (Foyer and Mullineaux, 1998; Smirnoff *et al*., 2000), partly catalysed by apoplastic ascorbate oxidase (Esaka *et al*., 1988; Lin and Varner, 1991; Li *et al*., 2017) and partly non‐enzymically (Green and Fry, 2005a). DHA can be further oxidised to a range of compounds, including *O*‐oxalyl‐threonate esters and free oxalate (OxA) (Green and Fry, 2005b; Parsons *et al*., 2011; Parsons and Fry, 2012). The oxidation of ascorbate to DHA is reversible in plants (Foyer and Halliwell, 1977), but further degradation of DHA represents a loss of vitamin C.

Non‐cyclic oxalyl‐threonate (OxT) is the predominant product formed during the *in‐vitro* oxidation of DHA by H_2_O_2_ (Parsons *et al*., 2011; Parsons and Fry, 2012). It was also detected in the apoplast of healthy rose cell‐suspension cultures (Green and Fry, 2005b), in tomato plants (Truffault *et al*., 2017) and in spinach leaves (Dewhirst *et al*., 2017). There are three isomers of OxT (2‐OxT, 3‐OxT and 4‐OxT), which are interconvertible non‐enzymically; at equilibrium *in vitro*, and also as detected naturally occurring *in vivo*, 4‐OxT predominates (Green and Fry, 2005b; Parsons *et al*., 2011; Parsons and Fry, 2012). In addition, DHA oxidation yields cyclic oxalyl‐threonate (cOxT), probably mainly 3,4‐cOxT (Green and Fry, 2005b). Oxidation of DHA by H_2_O_2_ gives OxT, cOxT and free OxA in the ratio ~6:1:1 (Parsons *et al*., 2011). *In vivo*, the oxidation products of DHA gradually undergo enzyme‐catalysed irreversible hydrolysis, cOxT → OxT → OxA + threonate (Green and Fry, 2005a; Parsons *et al*., 2011; Parsons and Fry, 2012), indicating the presence of oxalyl esterase activities.

The interconversion of oxalyl esters, e.g. 3‐OxT ↔ 4‐OxT, indicates intramolecular migration of oxalyl groups, and raises the possibility that intermolecular transfer of oxalyl groups may also occur by transesterification reactions, e.g.

OxT+ROH↔OxR+T

where ROH is an unspecified alcohol and T is threonate. This mechanism of synthesising oxalyl esters (OxR) could occur in the apoplast despite the lack of any conventional ‘energy source’ such as ATP or acyl‐CoA thioesters. OxT and ROH would be the oxalyl donor and acceptor substrates respectively. In place of ROH, the acceptor substrate could potentially be a thiol (RSH) or an amine (RNH_2_), yielding an oxalyl thioester or an oxalyl amide respectively. Transacylation is analogous to the transglycosylation processes that generate new saccharide structures in the cell wall, catalysed by transglycanases and transglycosidases (Franková and Fry, 2013).

The enzyme(s) responsible for oxalyl esterase activity in the apoplast (Green and Fry, 2005a,b) could potentially catalyse transacylation reactions in competition with hydrolysis, just as some glycanases and glycosidases can catalyse transglycosylation in competition with hydrolysis (Bojarová and Křen, 2009; Brás *et al*., 2010; Franková and Fry, 2011). The acceptor substrate, e.g. ROH, could potentially be any of a wide range of apoplastic solutes and cell‐wall structural components such as polysaccharides.

Plant cell walls are made up of cellulose microfibrils embedded in a matrix of hemicellulose and pectin, held in place by intermolecular cross‐links (Fry, 1986, 2017; Cosgrove, 2005; Scheller and Ulvskov, 2010), and are essential in conferring the cells’ shape, size and strength. They also provide a barrier to environmental stresses and pathogens (Bellincampi *et al*., 2014). Although the plant cell wall must be a strong, sometimes inextensible, structure, it is also dynamic and can continually remodel during cell growth (Bashline *et al*., 2014; Braidwood *et al*., 2014). Wall polysaccharides are subjected to numerous post‐synthetic modifications, often involving esterification. Some of these polysaccharide‐modifying reactions occur intraprotoplasmically before secretion [e.g. *O*‐acetyl‐esterification (Gille and Pauly, 2012), *O*‐feruloyl‐esterification (de Souza *et al*., 2018), oxidative feruloyl coupling (Fry *et al*., 2000), methyl‐esterification and ‐etherification (Kauss *et al*., 1967)], and borate diester cross‐linking (Chormova *et al*., 2014a,b); others occur in the wall itself after secretion [e.g. hydrolysis, transglycosylation and further oxidative feruloyl coupling (Burr and Fry, 2009; Franková and Fry, 2013)].

Interpolymeric covalent bonds proposed to contribute to wall architecture (Fry, 1986) include glycosidic xyloglucan–pectin bonds (Thompson and Fry, 2001; Popper and Fry, 2008), borate bridges between rhamnogalacturonan II domains (Fleischer *et al*., 1999; Pérez *et al*., 2003; O'Neill *et al*., 2004; Chormova *et al*., 2014a,b), dimeric, trimeric and larger oxidative coupling products of feruloyl polysaccharides (Fry *et al*., 2000; Burr and Fry, 2009; Harris and Trethewey, 2010), *O*‐uronoyl–sugar ester bonds (Brown and Fry, 1993; Carpita and Gibeaut, 1993; Marry *et al*., 2006) and *N*‐uronoyl–amine amide bonds (Perrone *et al*., 2002). Cross‐linking between polymers of adjacent cell walls would lead to cell adherence, and cross‐linking between components within a cell wall leads to a decrease in wall extensibility (Ishii, 1997). The present paper raises the interesting possibility of an ester‐based cross‐link, polysaccharide–oxalate–polysaccharide.

Plants possess many known acyltransferases (D'Auria, 2006), although enzymes catalysing trans‐oxalylation have not yet been reported. In many cases, the acyl donor substrate is a ‘high‐energy’ metabolite such as a coenzyme A (CoA) thioester, and the reaction is then essentially irreversible, with free CoA as by‐product. Examples are in the BAHD superfamily (D'Auria, 2006; de Souza *et al*., 2018) named after the first four such enzymes characterized: BEAT (benzylalcohol‐*O*‐acetyltransferase (Dudareva *et al*., 1998)), AHCT (anthocyanin‐*O*‐hydroxycinnamoyltransferase (Fujiwara *et al*., 1998)), HCBT (anthranilate *N*‐hydroxycinnamoyl/benzoyltransferase (Yang *et al*., 1997)) and DAT [deacetylvindoline‐4‐*O*‐acetyltransferase (St‐Pierre *et al*., 1998)]. In other cases, the donor substrate is not appreciably more ‘energetic’ than the product, and the reaction is reversible; for example, cutin synthase uses 2‐mono(10,16‐dihydroxy hexadecanoyl)glycerol as donor substrate and transfers the hydroxyacyl group to the nascent cutin polymer (Yeats *et al*., 2012). The oxalyltransferase reactions reported in the current study are likely to be reversible.

Acyltransferases have a wide range of functions in the plant, including in the modification of phenolic compounds (Cheynier *et al*., 2013). For example, the coumaroylation and sinapoylation of anthocyanins stabilises the blue colour of certain flowers (Luo *et al*., 2007).

The attachment of an aliphatic acyl group, palmitate, to cysteine residues of proteins associated with the tonoplast is thought to contribute to increased resistance to salt stress (Zhou *et al*., 2013). Palmitoyltransferases play a role in plant growth and development. Plants lacking in an *S*‐acyltransferase (known as AtPAT10, which acts to transfer an acyl group such as palmitate to a cysteine residue of an acceptor protein) were found to show reduced cell expansion and cell division, resulting in a dwarf phenotype (Qi *et al*., 2013). The growth of root hairs was found to be regulated by an *S*‐acyltransferase (called TIP1), responsible for controlling cell shape and growth (Hemsley *et al*., 2005). Equally, the development of pollen in rice was found to require a hydroxycinnamoyl:fatty acid acyltransferase reaction (Xu *et al*., 2017). Acyl sugars, produced from the esterification of sugars with fatty acids, are thought to protect plants from phytophagous insects (Chortyk *et al*., 1997; Weinhold and Baldwin, 2011). These acyl sugars are exuded from trichomes, and are especially common in the Solanaceae (Schilmiller *et al*., 2012).

Acyltransferases also play a role in cell‐wall modifications, including in the incorporation of hydroxycinnamates into poalean cell‐wall polysaccharides (Bartley *et al*., 2013; Mitchell *et al*., 2007; de Souza *et al*., 2018). Furthermore, the extraprotoplasmic components cutin and suberin, which act as barriers for pathogens and water, require acyltransferases during their synthesis (Beisson *et al*., 2007; Yeats *et al*., 2012).

**Figure 1:**
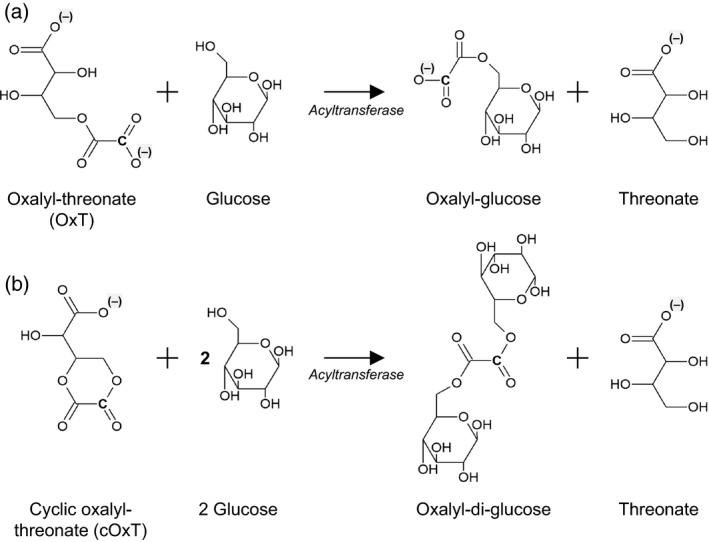
Proposed oxalyl‐sugar ester formation via acyltransferase activity. Oxalyl‐threonate (OxT) and cyclic oxalyl‐threonate (cOxT), which are *in‐vivo* oxidation products of vitamin C, are proposed to serve as oxalyl donor substrates with sugars (e.g. glucose, shown here) as acceptor substrates. The sugar could in principle be a residue of a wall polysaccharide. (a) Formation of an oxalyl‐sugar mono‐ester with OxT as donor substrate. (b) Hypothetical formation of a sugar–oxalyl‐sugar diester with cOxT as donor substrate. The radiolabelled carbon (derived from C‐1 of the [^14^C]ascorbate from which the [^14^C]OxT was produced) is shown by a bold C.

In the present paper, we demonstrate that oxalyl transfer reactions, with OxT and possibly cOxT as apoplastic donor substrates (Figure 1a), are post‐synthetic modifications that occur in the cell wall. We also explore the possibility that oxalate, being bifunctional, could cross‐link two polysaccharide molecules (Figure 1b). Such reactions add to the repertoire of wall modifications, and potentially covalent bonds, proposed to contribute to cell‐wall architecture.

## Results

### Transacylation with [^14^C]OxT as donor substrate in spinach cell‐suspension cultures *in vivo*

In preliminary work, we showed that radioactivity from [^14^C]DHA was incorporated into the cellular polymeric fraction (AIR) (Figure S1). However, DHA has diverse metabolic fates, forming cOxT, OxT and OxA, as well as diketogulonate and its downstream products (Kärkönen *et al*., 2017). We therefore focused on the incorporation of one specific naturally occurring DHA catabolite, OxT.

**Figure 2:**
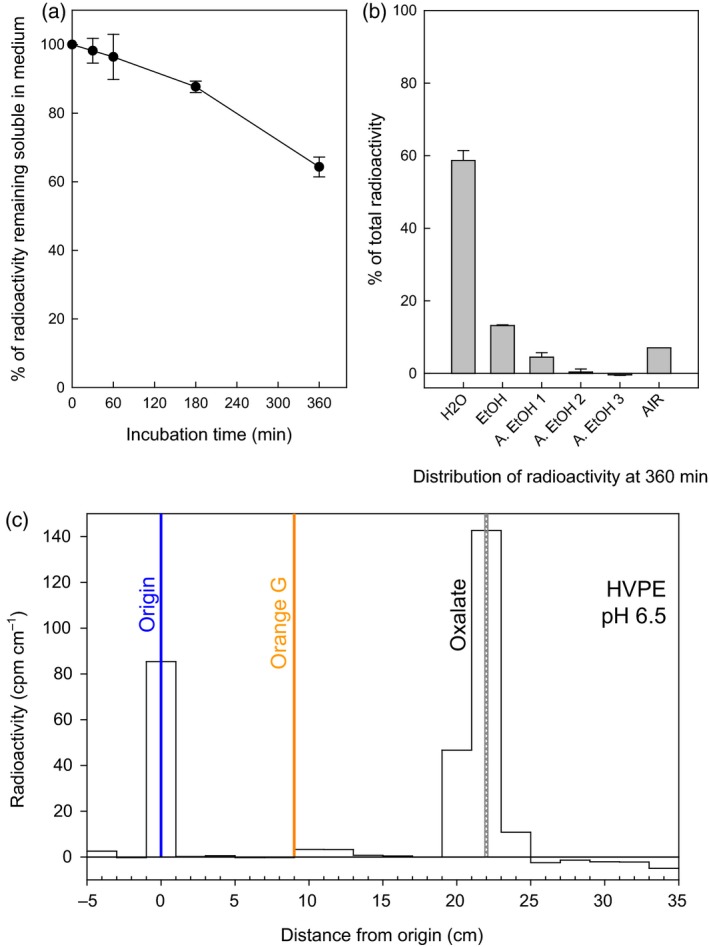
Oxalyl esters accumulate in alcohol‐insoluble residue of spinach cell cultures incubated with oxalyl‐threonate. Triplicate mini‐cultures of spinach (250 mg cells in 500 μl medium, 1 week after subculturing) were fed 0.67 μm [^14^C]oxalyl‐threonate. (a) Soluble radioactivity remaining in medium. Samples of cell‐free medium (50 μl), taken at intervals, were assayed for ^14^C. Each point is the mean of three replicate cultures ± standard error (SE). (b) Distribution of radioactivity at 360 min. After 6 h the remaining medium was removed and the cells were washed sequentially in H_2_O, ethanol (EtOH) and acidified ethanol (A EtOH); portions of these washes were assayed for ^14^C. The bar labelled ‘H_2_O’ represents the radioactivity present in the medium plus the water‐wash of the cells. The alcohol‐insoluble residue (AIR) left after the washes, comprising predominantly cell‐wall material, was also assayed. (c) The 360‐min radiolabelled AIR was alkali‐hydrolysed, acidified and electrophoresed at pH 6.5 in the presence of EDTA. The ^14^C profile is shown, together with the positions of markers (orange G and oxalate). [Colour figure can be viewed at http://www.wileyonlinelibrary.com].

**Figure 3:**
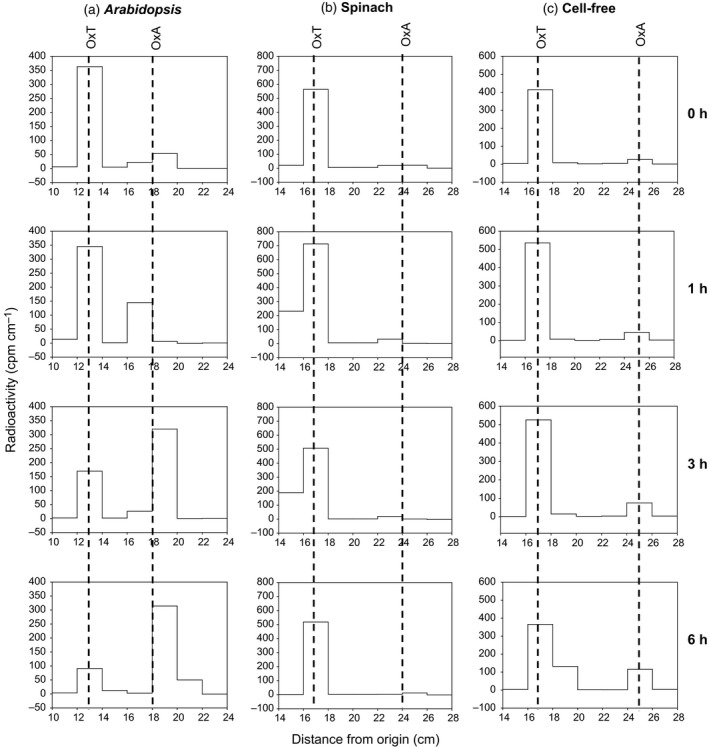
Fate of [^14^C]oxalyl‐threonate in Arabidopsis and spinach cell culture. Samples of culture medium (50 μl) collected from spinach or Arabidopsis cell mini‐cultures (250 mg cells plus 500 μl medium) or pH 4.5 buffer (cell free) that had been incubated with 0.67 μm [^14^C]OxT for 0–6 h were fractionated by electrophoresis at pH 6.5. Vertical lines indicate the positions of authentic markers oxalyl‐threonate (OxT) and oxalic acid (OxA).

To investigate the possible transfer of oxalyl groups from OxT to cell‐wall components, representing a mechanism of wall modification, we fed OxT (^14^C‐labelled in the oxalyl moiety) to living spinach cell‐suspension cultures. About 26% of the ^14^C was removed from solution in the apoplastic volume (culture medium) during a 6‐h incubation (Figure 2a). Most of the ^14^C was recovered in a water‐wash at 6 h (Figure 2b), and was found to be still in the form of [^14^C]OxT (Figure 3b). In a similar experiment with Arabidopsis cells, the [^14^C]OxT was gradually hydrolysed to [^14^C]OxA (Figure 3a), indicating greater oxalyl esterase activity in Arabidopsis. For this reason, we focused on the spinach culture. A minority of the ^14^C fed to spinach cultures was found in the ethanol washes, which represent intraprotoplasmic solutes (Figure 2b).

After repeated washings with neutral and acidified ethanol, ensuring that essentially all low‐M_r_ compounds had been removed, some ^14^C remained in the spinach alcohol‐insoluble residue (AIR), which represents high‐M_r_ material, predominantly cell‐wall polysaccharides. AIR is a widely adopted preparation of total cellular polymers based on the fact that essentially all small biochemicals (M_r_ < 1000) are soluble in 70% ethanol whereas polysaccharides and most other cellular polymers are not (Selvendran, 1975). The radioactivity in AIR potentially indicates the formation of [^14^C]oxalyl esters with wall components.

The radiolabelled AIR was treated with NaOH, which saponifies (hydrolyses) ester bonds. The release of free [^14^C]OxA after saponification (Figure 2c) supports the predicted formation of an oxalyl‐polysaccharide ester. Some radioactivity remained insoluble after saponification, and this material remains unidentified.

### Formation of oxalyl‐sugar esters in spinach cell‐suspension culture

As AIR consists predominantly of wall polysaccharides, we hypothesised that the acceptor substrate for the [^14^C]oxalyl residue would be a carbohydrate. To investigate this hypothesis, we incubated [^14^C]OxT with spinach cell culture, or cell‐free culture filtrate, in the presence of various additional carbohydrates as exogenous acceptor substrates. Each carbohydrate was supplied at 5% (w/v): this approach is an approximation to equalising the molar concentrations of –OH groups, which are the functional group serving as the acceptor substrate. For example, at a constant 5% (w/v), xylose, glucose, sucrose and raffinose solutions contain about 1.3, 1.4, 1.2 and 1.1 M –OH groups respectively.

**Figure 4:**
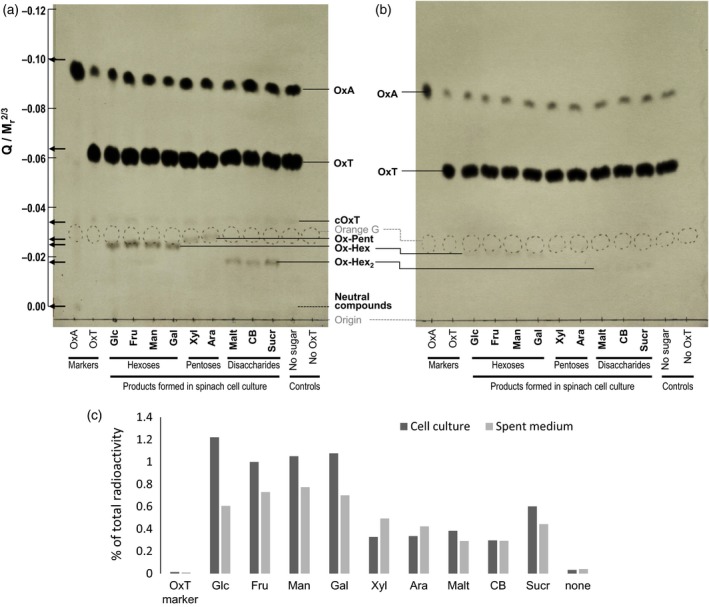
Formation of oxalyl‐sugars in spinach cell culture and spent culture medium with [^14^C]oxalyl‐threonate plus sugars. (a–c) Aliquots (10 μl) of spinach cell culture, including cells (a) or excluding cells (b), were incubated with 50 μm [^14^C]OxT plus various sugars (5%, w/v) for 16 h. Each sample was then electrophoresed at pH 6.5 in the presence of EDTA, and autoradiographed. (c) Yield of [^14^C]oxalyl‐sugars, quantified by scintillation counting. Ara, arabinose; CB, cellobiose; cOxT, cyclic oxalyl‐threonate; Fru, fructose; Gal, galactose; Glc, glucose; Malt, maltose; Man, mannose; OxA, oxalic acid; Ox‐Hex, oxalyl‐hexose; Ox‐Hex_2_, oxalyl ester of a hexose disaccharide; Ox‐Pent, oxalyl‐pentose; OxT, oxalyl‐threonate; Sucr, sucrose; Xyl, xylose. The scale to the left of (a) indicates the estimated ratio of the ions’ charge (Q) to M_r_^2/3^ (i.e. molecular weight to the power of ⅔). [Colour figure can be viewed at http://www.wileyonlinelibrary.com].

Transacylation products – soluble extracellular [^14^C]oxalyl‐sugars – were indeed produced by the culture, as indicated by radioactive spots with the predicted mobilities (Figure 4a). The yield was 0.3–1.2% of the total ^14^C. Electrophoretic mobility is proportional to the *Q*:*M*_r_^⅔^ ratio (Offord, 1966; Fry, 2011) where *Q* is the net charge of the molecule (at the pH of the electrophoresis buffer) and *M*_r_ to the power of ⅔ is an indication of its relative surface area. The *Q*:*M*_r_^⅔^ ratios of OxA, OxT, cOxT, oxalyl‐pentoses, oxalyl‐hexoses and oxalyl‐disaccharides, calculated on the basis that all −COOH groups are fully ionised at pH 6.5, are shown on Figure 4(a) (arrows on the left‐hand scale); the distribution of radioactive bands is highly compatible with their proposed structures. The highest yields of radioactive products were obtained when the acceptor substrates were hexoses (the ketose fructose slightly exceeding the aldoses glucose, mannose and galactose), less product was formed from sucrose, less still from cellobiose and maltose, and least from the pentoses (xylose and arabinose). These relative yields may be explained in terms of:

- the different molar concentrations of the acceptor substrates used (all sugars were supplied at 5% w/v, thus 146 mm hexose disaccharide, 278 mm hexose monosaccharide and 333 mm pentose monosaccharide), which would predict an order of effectiveness pentose > hexose > disaccharide; partially over‐ridden by:
- the number of primary alcohol (−CH_2_OH) groups per molecule [<0.05 for the pentoses (as arabinose and xylose are <5% and <1%, respectively, in the furanose form, which has a −CH_2_OH group (Collins and Ferrier, 1995)), ˜1 for the aldoses, ˜1.3 for fructose, two for maltose and cellobiose, and three for sucrose].

The results indicate that the preferred acceptor substrate site is the primary −OH group present in some sugars, but that secondary −OH groups (e.g. in xylose) can serve to a limited extent.

The yield of oxalyl‐sugar esters was generally slightly higher in the presence of cells than in spent medium (Figure 4b,c). This suggests that the transfer of an oxalyl group depends on acyltransferases that are at least partly attached to the spinach cell surface rather than dissolved in the culture medium.

The acyltransferase activity of washed spinach cells was destroyed by boiling (Figure S2), supporting the conclusion that it is attributable to an enzyme. As expected, the yield of the enzymic reaction product was time‐dependent (Figure S3).

The apparent optimum pH for production of [^14^C]oxalyl‐glucose (OxG) during a 16‐h incubation with washed spinach cells was about 6 (Figure S4). At pH 7 there was a greater yield of free [^14^C]oxalate, but a lower yield of OxG. However, a similar assay conducted for shorter incubation times showed that the true optimum pH for [^14^C]OxG production was higher, at least pH 7.5 (Figure S5). By 16 h, the hydrolase activity of the enzyme(s) may have depleted the OxG initially formed by the acyltransferase activity.

### Formation of oxalyl‐sugar esters by spinach cell‐wall enzyme extract

**Figure 5:**
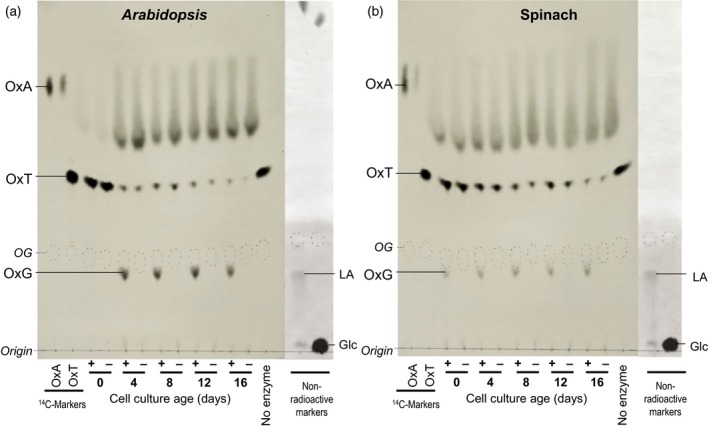
Acyltransferase time courses for enzymes extracted from Arabidopsis and spinach. (a, b) Cell‐wall enzymes were salt‐extracted from live cell cultures of (a) Arabidopsis and (b) spinach at various ages after subculturing, then dialysed, freeze dried and redissolved at 1% (w/v) in 10 mm PIPES (Na^+^, pH 7.0). The enzyme preparations (10 μl) were incubated with 50 μm [^14^C]oxalyl‐threonate (OxT) with (+) or without (−) 5% (w/v) glucose for 4 h. Products were separated by electrophoresis at pH 6.5 and autoradiographed (OxG, oxalyl‐glucose). Non‐radiolabelled markers (Glc, glucose; LA, lactobionic acid) were stained in silver nitrate. LA is a readily silver‐stainable compound possessing 12 C atoms and 1^−^ charge, and is thus a useful nearby‐migrating marker for substances such as oxalyl‐disaccharides (14 C atoms, 1^−^). Radioactive markers: OxA, oxalate; OxT, oxalyl‐threonate. Pencilled: OG, orange G. [The streaking of the [^14^C]oxalate spots in Figures 5 and 6 (compare with Figure 4) was caused by omission of EDTA from the electrophoresis buffer. This streaking does not interfere with interpretation of the results.] [Colour figure can be viewed at http://www.wileyonlinelibrary.com].

Salt‐extractable (thus ionically bound) wall enzymes from spinach and Arabidopsis cultures were found to have acyltransferase activity. This was indicated by the formation of oxalyl‐glucose (OxG) when the extracts were incubated with [^14^C]OxT plus glucose (Figure 5).

Wall enzymes extractable from 0‐day cells (immediately after subculture) were the least active, especially in Arabidopsis, but the activity was very evident by 4 days (Figure 5). Between 4 and 16 days after subculturing there was little consistent change. We tested 12‐day and 16‐day cultures, which did not differ from each other and thus we assume 14‐day cultures were also identical. The ‘old’ cultures can thus be contrasted with 0‐day cultures, which are actually 14‐day cultures that have been recently plunged into fresh medium.

In the absence of acceptor substrate (glucose), the OxT was partially hydrolysed to OxA. The presence of glucose resulted in a pronounced shift towards an OxT → OxG reaction at the expense of the OxT → OxA reaction (more clearly visible in Figure 5(a), in which OxG makes up a higher proportion of the total reaction products). The data are quantified in Figure S6. This result indicates acyltransferase and acylhydrolase activities as competing reactions. Further data, quantifying the reciprocity between OxG and OxA products in response to increasing acceptor substrate (glucose) concentrations, are shown in Figure S7.

### Oxalyl ester formation with alternative donor and acceptor substrates

**Figure 6:**
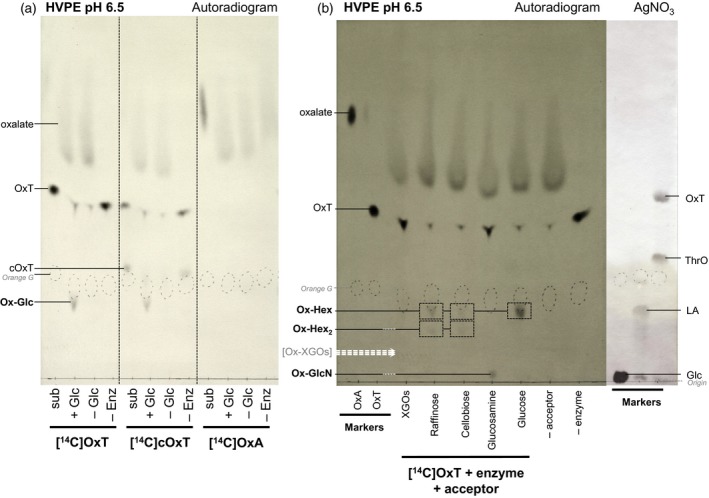
Oxalyl‐sugar formation *in vitro* with various donor and acceptor substrates *(a) Three potential donor substrates with glucose as acceptor. Arabidopsis cell‐wall enzyme (1% w/v) was incubated with 50 μm [^14^C]OxT, [^14^C]cOxT or [^14^C]OxA, plus 5% (w/v) glucose (final volume 10 μl). Controls lacked glucose and/or enzyme. Products formed after 4 h were electrophoresed, then autoradiographed. Lanes labelled ‘sub’ (substrate) represent time‐0 samples.* (b) Five potential acceptor substrates with oxalyl‐threonate as donor. Incubations as in (a) with 50 μm [^14^C]OxT plus 5% (w/v) of the potential acceptor substrate. After 4 h, products were analysed as in (a). Controls lacked acceptor substrate and/or enzyme. Non‐radioactive markers were stained in AgNO_3_. White arrows show the predicted electrophoretic mobilities of oxalyl‐XGOs (from top to bottom: Ox‐XXXG; Ox‐XXLG; Ox‐XLLG) relative to those of Ox‐Hex_2_ and glucose, as estimated from Q:M_r_^2/3^ values (see Figure 4). OxT, oxalyl‐threonate; cOxT, cyclic oxalyl‐threonate; Ox‐Glc, oxalyl‐glucose; Ox‐Hex, oxalyl‐hexose; Ox‐Hex_2_, oxalyl ester of a hexose disaccharide; Ox‐GlcN, *O*‐oxalyl‐glucosamine; ThrO, threonate; LA, lactobionate; Glc, glucose; XGOs, xyloglucan oligosaccharides. [Colour figure can be viewed at http://www.wileyonlinelibrary.com].

OxT had been used as the donor substrate for the above experiments, but cOxT might also be a suitable oxalyl donor, and has the interesting potential ability to link a single oxalyl group via two ester bonds, forming a cross‐link [e.g. sugar–oxalate–sugar; Figure 1b]. We therefore compared cOxT and OxT as donor substrates, and OxA as a control (Figure 6a).

Free OxA cannot serve as an oxalyl donor as it is not activated; indeed, as expected, incubation of glucose with OxA in the presence of an Arabidopsis acyltransferase extract did not produce OxG (Figure 6a). However, incubation of the acyltransferase preparation and glucose with either OxT or cOxT did produce OxG (Figure 6a), suggesting that cOxT might be an effective donor substrate. However, all preparations of cOxT also contained a proportion of OxT so it was not possible to determine whether the oxalyl group transferred to glucose originated directly from cOxT. The intensity of both cOxT and OxT spots decreased in the presence of the enzyme, which could indicate the occurrence of:

cOxT+glucose→OxG+OxT

followed by

OxT+glucose→OxG+threonate,

and/or

$$\text{cOxT}+H_{2}O\to \text{OxT}$$

followed by

OxT+glucose→OxG+threonate.

The theoretical cross‐linked diester formed from cOxT in the reaction shown in Figure 1(b) (glucose–oxalyl‐glucose) would have no charge, and would appear in the neutral area (co‐migrating with free glucose) after high‐voltage paper electrophoresis (HVPE). There is no such ^14^C‐labelled compound visible (Figure 6a), suggesting that cOxT did not appreciably function to cross‐link glucose molecules. There is also no evidence of the theoretical intermediate, threonate–oxalyl‐glucose, formed as:

$$\begin{array}{cc} & \text{cOxT}+\text{glucose}\to \text{threonate}-\;\text{oxalyl‐glucose;}\\ & \text{threonate}-\;\text{oxalyl‐glucose}+\text{glucose}\to \\ & \text{glucose}-\;\text{oxalyl‐glucose}+\text{threonate,} \end{array}$$

which with 12 carbon atoms and one negative charge (C_12_, 1^−^), would have an electrophoretic migration rate similar to that of lactobionic acid [C_12_, 1^−^; Figure 6b] or oxalyl‐cellobiose [C_14_, 1^−^; compare with Figure 4a].

A wider selection of sugars was tested for their suitability as acceptor substrates in the oxalyl transfer reaction catalysed by the Arabidopsis enzyme extract (Figure 6b), including a mixture of xyloglucan oligosaccharides (XGOs), raffinose (a trisaccharide of galactose, glucose and fructose), cellobiose and glucosamine. The radioactive product obtained when glucosamine was tested as the acceptor substrate was a neutral compound (Figure 6b); therefore this product must contain both the amine group and an oxalyl group. This result indicates that the oxalyl group became *O*‐linked to the glucosamine, probably at the primary hydroxy group on C‐6. The alternative would have been for the oxalyl group to become *N*‐linked to the glucosamine through a secondary amide bond, but the product in this case would have a net negative charge.

Seven additional amines were also tested as potential acceptor substrates and proved ineffective (Figure S8). Therefore the acyltransferase activity was not capable of forming oxalyl‐amides under conditions suitable for formation of an oxalyl‐glucose (OxG) ester.

Incubation of OxT with XGOs in the presence of acyltransferase did not yield any detectable oxalyl ester compounds (Figure 6b). Oxalyl‐XGOs would have a mobility lower than that of OxG, as they have a larger mass and one negative charge. The compounds would run in the area between the origin and orange G on the electrophoretogram (dashed arrows in Figure 6b), but this area appears empty, showing that any such compounds were below the limit of detection.

The reaction of OxT with raffinose and cellobiose in the presence of an acyl transferase produced two compounds corresponding to an oxalyl‐hexose and an oxalyl‐disaccharide (Figure 6b). As raffinose is a trisaccharide, the production of these compounds suggests that an enzyme present in the Arabidopsis cell‐wall extract quickly hydrolysed the raffinose to a monosaccharide plus a disaccharide. Equally, the production of OxG from cellobiose indicates β‐glucosidase activity in the Arabidopsis enzyme extract (Figure 6b).

To investigate the possibility of polysaccharides acting as acceptor substrates, we tested polysaccharide–paper complexes as potential oxalyl acceptor substrates, with [^14^C]OxT as donor and an Arabidopsis enzyme extract containing the acyltransferase. The products were washed repeatedly in ethanol. If the acyltransferase had transferred the oxalyl group from [^14^C]OxT to the polysaccharide–paper complex, then the radioactivity would have become covalently bound to the complex as an ethanol‐insoluble [^14^C]oxalyl‐polysaccharide ester.

**Figure 7:**
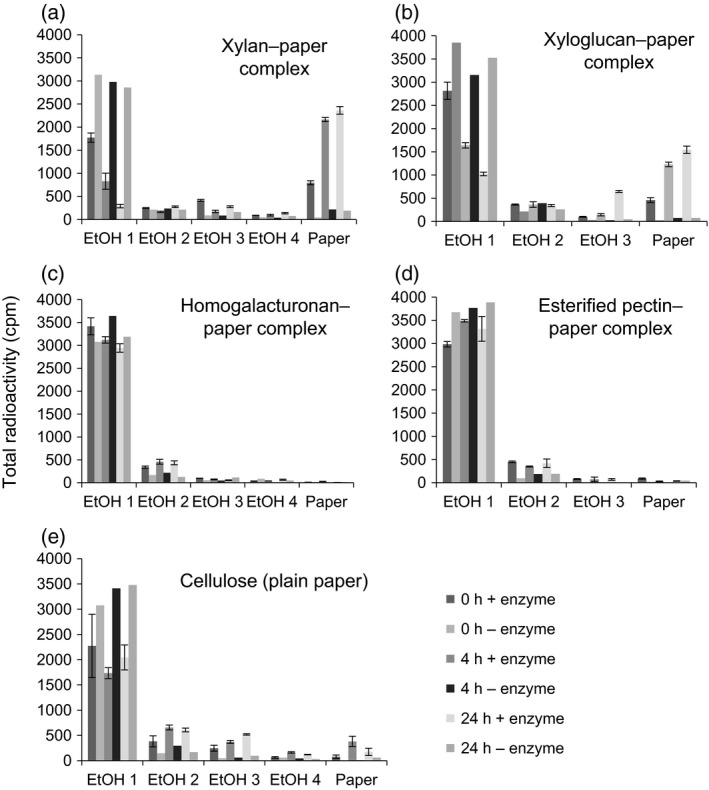
Formation of oxalyl esters with polysaccharide–cellulose complexes as acceptor substrates catalysed by Arabidopsis acyltransferase. Polysaccharide–cellulose complexes (polysaccharide‐impregnated paper; 1.5 × 2.0 cm) were incubated with Arabidopsis enzyme extract plus [^14^C]oxalyl‐threonate at pH 7 (total liquid volume 35 μl) for 0–24 h. Controls lacked enzyme. (a–e) The polysaccharides tested were: (a) xylan, (b) xyloglucan, (c) homogalacturonan, (d) methyl‐esterified pectin, or (e) untreated paper (i.e., cellulose only). After the specified incubation times the papers were washed in 70% ethanol three or four times, and then the radioactivity present in the washes and in the final washed paper was assayed. Each sample containing enzyme was in triplicate, and the enzyme‐free controls were single samples.

Very little radioactivity was incorporated into plain paper (Figure 7e), showing that oxalyl‐cellulose ester bonds were not formed. However, the hemicellulose–paper complexes, with either xylan (Figure 7a) or xyloglucan (Figure 7b), showed appreciable incorporation of radioactivity into the polysaccharide–paper complex, increasing up to at least 24 h. Controls containing no enzyme extract showed very little radioactivity remaining in the paper after ethanol washing. This result indicates that Arabidopsis acyltransferase activities had formed ester bonds between the oxalyl group (derived from OxT) and the hemicelluloses.

Conversely, the pectin–paper complexes, with either homogalacturonan (Figure 7c) or methyl‐esterified pectin (Figure 7d), supported no incorporation of radioactivity. This result shows that the pectic polysaccharides did not act as oxalyl acceptor substrates for acyl transferase(s).

### Oxalyl‐glucose stability in spinach cell culture

**Figure 8:**
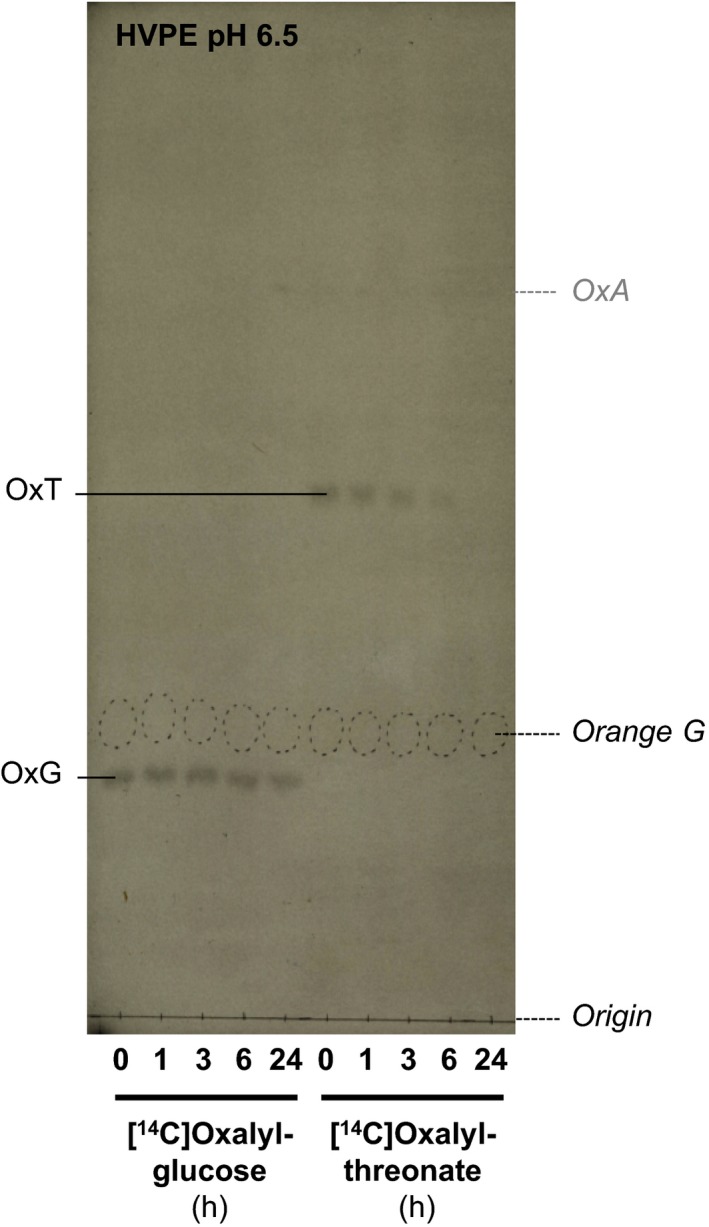
[^14^C]Oxalyl‐glucose is stable in spinach apoplast. Spinach cell mini‐cultures [250 mg cells (harvested 7 days after subculture) plus 500 μL of 7‐day medium medium] were incubated with ~0.67 μm [^14^C]oxalyl‐glucose (OxG) or [^14^C]oxalyl‐threonate (OxT). After 0–24 h, samples of the culture medium were electrophoresed at pH 6.5 (in the presence of 5 mm EDTA) and autoradiographed. The internal marker orange G spots are circled in pencil, and the expected position of oxalic acid (OxA; not clearly visible) is marked in grey. [Colour figure can be viewed at http://www.wileyonlinelibrary.com].

The potential biological roles of oxalyl‐carbohydrate esters depend on their longevity *in vivo*. We therefore tested the fate of two relevant [^14^C]oxalyl esters in the presence of living cells. Spinach cell‐suspension cultures were fed 0.67 μm [^14^C]OxT or [^14^C]OxG and the culture medium was sampled at intervals (Figure 8). Extracellular OxT remained almost stable in the presence of living cells for 6 h, although it had gone from the culture medium by 24 h. The major hydrolysis product should be [^14^C]OxA, but negligible free [^14^C]OxA was found (Figure 8); it may have been precipitated as its insoluble calcium salt and thus lost from the medium.

OxG, conversely, was largely stable for the whole 24‐h incubation period (Figure 8). The intensity of the band of OxG did not appreciably diminish over time, suggesting that this compound is not absorbed or adsorbed by the spinach cells, nor hydrolysed extracellularly.

## Discussion

OxT, a major product formed from vitamin C via two oxidation steps, ascorbate → DHA (via ascorbate free radical) and DHA → OxT, largely remained in the apoplastic space of spinach cell‐suspension cultures rather than entering the protoplasm. This finding contrasts with the behaviour of DHA, which does enter the protoplasm (Horemans *et al*., 2008). OxT was only slowly hydrolysed to free OxA in the medium (apoplast) of spinach cell cultures, showing that spinach cells do not secrete appreciable activities of oxalyl esterase into the apoplast. This result was not the case in Arabidopsis cultures, in which OxT was quickly hydrolysed to OxA (and presumably non‐radioactive threonate). Rose cell‐suspension cultures have also been demonstrated to possess oxalyl esterase activity which can hydrolyse OxT in the apoplast (Green and Fry, 2005b).

Evidence was obtained for the transfer of the oxalyl group from [^14^C]OxT to components of the spinach cell wall (AIR). The radiolabelled moiety was released in the form of free [^14^C]OxA upon saponification, indicating that it had become esterified to wall material. The addition of oxalyl side‐chains onto cell‐wall polymers represents a wall modification, adding to the repertoire of modifications that wall polysaccharides undergo, such as *O*‐acetylation, methylation, methylesterification (Schmelter *et al*., 2002; Pettolino *et al*., 2012), feruloyl esterification (Burr and Fry, 2009) and borate bridging (Chormova *et al*., 2014a,b).

As a simplified model reaction in which to explore the oxalyltransferase activity, we fed living spinach cells with [^14^C]OxT plus low‐*M*_r_ sugars. Products were [^14^C]oxalyl‐sugar esters, identified by their electrophoretic mobilities. Such oxalyl‐sugars were formed in lower amounts in cell‐free spent culture medium, suggesting that the enzyme (acyltransferase) responsible for this activity was predominantly bound to the cell walls rather than free in the apoplast.

Salt‐extractable cell‐wall enzymes from spinach and Arabidopsis cell cultures showed high acyltransferase activity, determined by the formation of radiolabelled oxalyl‐sugars from [^14^C]OxT plus non‐radioactive sugars. This demonstrates that at least some of the enzyme(s) were ionically bound to the cell wall.

It is interesting that wall enzymes eluted with salt from day‐0 Arabidopsis cultures immediately after sub‐culturing had almost no acyltransferase activity (Figure 5a), despite these cells being equivalent to a 14‐day‐old culture (the approximate age at which the cells were sub‐cultured, by dilution into fresh medium). This result was repeated in a replicate experiment. Some cells are known to react to stress by oxidatively cross‐linking certain proteins in the cell wall (Otte and Barz, 1996; Brady and Fry, 1997) rendering them salt‐inextractable. It is possible that plunging the Arabidopsis cells suddenly into fresh medium elicited a stress response, rendering the acyltransferase(s) inextractable. This would not necessarily mean the acyltransferase was inactive or absent from these cells, merely inextractable with 1 m NaCl. The spinach cell‐wall extracts showed a similar trend (Figure 5b), although not as pronounced as in Arabidopsis.

We tested 17 carbohydrates as potential acceptor substrates [nine different sugars in Figure 4(a), a further three in Figure 6(b), and five polysaccharides in Figure 7]. The preferred acceptor substrates for the acyltransferase reaction seemed to be those with readily accessible primary −OH groups, e.g. on C‐6 of glucose and on C‐1 and C‐6 of fructose. The product formed from the reaction of [^14^C]OxT with glucosamine had no net charge, indicating that the oxalyl group was transferred onto an −OH group (probably at C‐6), rather than to the −NH_2_ group at C‐2.

Hemicelluloses also served as oxalyl acceptor substrates. Acyltransferase activity was able to transfer [^14^C]oxalate groups from OxT to xylan or xyloglucan – possibly reflecting processes that occur within the plant cell wall, adding oxalyl side‐chains to architectural polysaccharides.

As the donor substrate for these transacylations was OxT, containing only one ester group, then only a single ester linkage could be formed from each oxalate moiety to a cell‐wall component, resulting in the addition of an oxalyl side‐chain (Figure 1a). An alternative possibility would arise with cOxT as donor substrate, which could theoretically allow the formation of oxalyl diester cross‐links between two cell wall components, for example two polysaccharides. A simple model of such a cross‐linked structure would be glucose–[^14^C]oxalate–glucose (Figure 1b), which would be non‐ionic and therefore immobile on HVPE. We did not obtain evidence for such cross‐link formation in the present work (Figure 6a), perhaps owing to the difficulty of purifying [^14^C]cOxT, which partially hydrolyses to [^14^C]OxT during elution from dried preparative electrophoretograms despite being stable enough to migrate as a discrete spot during the electrophoresis process itself (Green and Fry, 2005b; Parsons and Fry, 2012). The contamination of cOxT by OxT is seen in Figure 6(a). Further work on the donor substrate capabilities of purified [^14^C]cOxT would be valuable.

Numerous acyltransferase activities have been detected in plants, but the transfer of an oxalyl group via the action of an acyltransferase has not to our knowledge previously been reported. Decorating wall polysaccharides with oxalyl groups may represent an interesting role of the wall‐bound acyltransferase activity reported here, and therefore also (working upstream) of:

- the apoplastic metabolites OxT and cOxT (oxalyl donor substrates), and thus
- their direct precursor, DHA, and thus
- ascorbate oxidase, the apoplastic enzyme that converts ascorbate to DHA, and thus
- the ascorbate secretion and DHA reabsorption systems.

High ascorbate oxidase activity often correlates with rapid cell expansion [e.g. in cowpea roots (Reid, 1941), maize roots and mesocotyls (Mertz, 1961, 1964), pumpkin cell‐suspension cultures (Esaka *et al*., 1988), courgette leaves and fruits (Lin and Varner, 1991), cultured tobacco cells (Kato & Esaka, 1999) and cotton trichomes (Li *et al*., 2017)], an observation that has been difficult to rationalise if viewed in terms of the enzyme's ability to destroy ascorbate, which is widely regarded as a ‘beneficial’ antioxidant associated with vitality and growth. Lin and Varner (1991) proposed the alternative view – that the key effect of ascorbate oxidase should be viewed as beneficially generating DHA rather than as detrimentally destroying ascorbate. They proposed three mechanisms by which apoplastic DHA, produced by ascorbate oxidase, might favour cell expansion:

- DHA may react with the ε‐amino groups of lysine residues of wall proteins, thus diminishing their ability to form wall‐tightening covalent (Schiff base) cross‐links with polysaccharides;
- DHA may complex with the guanidinium group of arginine residues, thus diminishing their ability to form wall‐tightening ionic bonds with acidic polysaccharides;
- DHA may be oxidised to free oxalic acid, which would chelate Ca^2+^ from homogalacturonan, thus cleaving pectin^.^Ca^2+^ ‘egg‐box’ structures and loosening the cell wall.

In the light of the present work, we now add a further suggestion – that DHA, as the precursor of OxT and cOxT, enables the addition of oxalyl ester side‐chains, and potentially oxalyl diester cross‐links, to hemicelluloses such as xylans and xyloglucans, therefore contributing to wall architecture. The natural occurrence, regulation and roles of oxalyl‐polysaccharide esters therefore now deserve detailed investigation.

The *O*‐oxalyl‐sugars and *O*‐oxalyl‐polysaccharides produced by the acyltransferase activity now reported have apparently not been detected in plants, or even described in the chemical literature. Here, besides demonstrating their production by plant enzymes *in vitro*, we effectively provide evidence for their biological occurrence: we infiltrated into the apoplast traces of radiolabelled DHA or OxT, slightly supplementing the natural DHA and OxT that are known to be present in the apoplast *in vivo* (Green and Fry, 2005b; Truffault *et al*., 2017). As the exogenous radiolabelled DHA and OxT were present at very low tracer concentrations, their metabolic fate must faithfully report the fate of the same (naturally occurring, unlabelled) endogenous compounds in living cultures.

*O*‐Oxalyl‐glucose was found to be relatively stable in living spinach cell cultures, remaining unhydrolysed in the apoplast, and it is likely that *O*‐oxalyl‐polysaccharides would also be stable. Thus, although OxT and cOxT are metabolically labile *in vivo*, undergoing hydrolysis and serving in acyltransferase reactions, their *O*‐oxalyl‐carbohydrate transacylation products have considerable longevity and could be proposed to serve lasting structural roles in cell‐wall architecture. The oxalate group is strongly acidic (i.e., has a very low *p*K_a_) and would act as a hydrophilic side‐chain that would remain anionic at all physiological pH vales and that could potentially influence the physical properties of wall polysaccharides. Other potential biological roles of oxalyl‐sugars could be as signalling molecules. In addition, their occurrence *in vivo* may be taken as a fingerprint, diagnosing the natural oxidation of apoplastic DHA. For all these reasons, the natural occurrence and biological roles of such compounds *in vivo*, and of the acyltransferases which catalyse their formation, warrants further investigation. We hope that this publication will stimulate such further work.

## Experimental procedures

### Maintenance of spinach and Arabidopsis cell‐suspension cultures

Spinach (*Spinacia oleracea* L., cv. Monstrous Viroflay) cell‐suspension cultures (Dalton and Street, 1976) were maintained in Murashige and Skoog basal salt (4.4 g/L, Sigma M‐5524) containing 1% (w/v) glucose; pH adjusted to 4.4 with NaOH. *Arabidopsis thaliana* cell‐suspension cultures were maintained in May and Leaver (1993) medium with 2% (w/v) glucose in place of sucrose. For both species, 180 ml of culture was grown in 500‐ml conical flasks under moderate constant light (25 μmol m^−2^ sec^−1^) at 25°C with shaking (100–115 rpm) and sub‐cultured every 2 weeks by eight‐fold dilution.

### Purification of ^14^C‐labelled compounds

l‐[1–^14^C]Ascorbic acid (16 kBq, 0.40 MBq/μmol; GE Healthcare, Amersham, UK) was treated with H_2_O_2_ (2 mol H_2_O_2_ per mol ascorbate, permitting a 4‐electron oxidation sequence, to yield the oxidation level of OxT) in a final volume of 60 μl for 30 min, then electrophoresed on Whatman 3mm paper in pH 6.5 buffer (pyridine/acetic acid/H_2_O, 33:1:300 v/v/v containing 5 mm EDTA) at 2.5 kV for 30 min (Fry, 2011). The paper was autoradiographed on Kodak Biofilm for 5 days. The strips corresponding to the ^14^C‐labelled compounds of interest (OxT or cOxT) were excised, and the compounds eluted in H_2_O by the Eshdat and Mirelman (1972) method.

Purified [^14^C]OxG was eluted from electrophoretograms similar to that shown in Figure 5(a) and concentrated *in vacuo*.

[^14^C]DHA was produced by ascorbate oxidase (from *Cucurbita* species; 1 U μl^−1^) treatment of [1‐^14^C]AA, in 10 mm formate buffer (pyridinium^+^, pH 5) and purified on a Dowex 1 anion‐exchange chromatography column, previously washed in (sequentially) 0.5 M NaOH, 0.5 m formic acid, 2 m sodium formate and 10 mm formate (pyridinium^+^, pH 5). The [^14^C]DHA was eluted in H_2_O.

### Fate of OxT, cOxT and OxG in living cell‐suspension cultures

Spinach or Arabidopsis cell‐suspension culture (7 days old, unless otherwise stated) was filtered on four layers of Miracloth (Calbiochem), then triplicate mini‐cultures [each 250 mg (fresh weight) of cells resuspended in 500 μl of 7‐day culture medium in flat‐bottomed glass vials] were shaken at ~120 rpm in constant light for at least 1 h before the addition of [^14^C]OxT or [^14^C]OxA or [^14^C]OxG (~200 Bq, in 1–5 μl) at ‘time 0’, to give a concentration of ~0.67 μm.

Samples of culture medium (50 μl) were taken in triplicate at time points and stored at −80°C until further analysis. For analysis of ^14^C incorporated into the cells, the remaining culture medium was removed, and the cells were washed sequentially in H_2_O, 70% ethanol, and three times in acidified ethanol (75% ethanol with 5% formic acid). For each wash, the cells were incubated in 5 ml of the solvent, in a 15‐ml tube, rotating on a wheel at 20°C for 20 min, followed by centrifugation for 10 min at 2000 ***g***; each wash and the final alcohol‐insoluble residue (AIR) were collected. The AIR was either dried for further analysis or suspended (in H_2_O or ethanol) and mixed with scintillation fluid (2 ml): either commercial ScintiSafe or a solution of 2,5‐diphenyloxazole (PPO; 3.3 g/L) and 1,4‐bis(5‐phenyl‐2‐oxazolyl)benzene (POPOP; 0.33 g/L) in toluene/Triton X100 (2:1, v/v). Radioactivity was quantified on a Beckman LS6500CE scintillation counter.

For hydrolysis of ester bonds, dried AIR (25 mg) was incubated in NaOH (0.1 m; 100 μl) for 1 h. The saponification reaction was stopped by the addition of excess acetic acid (0.2 m; 100 μl). The entire sample, including the insoluble fraction was loaded onto Whatman 3mm paper along with an internal marker of orange G. Markers of [^14^C]OxA and non‐radiolabelled glucose were also loaded. The paper was run by HVPE (Fry, 2011) at pH 6.5. After drying the paper was cut into strips, which were assayed for radioactivity bzy scintillation counting in 2 ml ScintiSafe.

### Eluting cell‐wall enzymes from plant cell‐suspension cultures

Cell wall proteins (including enzymes) were eluted from spinach or Arabidopsis cell cultures 7 days after subculturing, unless otherwise stated. Spent culture medium (200 ml) was filtered through four layers of Miracloth and the culture filtrate was stored at −80°C. The remaining cells were rinsed in 500 ml H_2_O, then resuspended in 100 ml 1 m NaCl in 5 mm succinate (Na^+^, pH 5.0) and shaken for 1 h at 4°C. The suspension was then filtered as before and 100 ml of the eluate was freed of salt by dialysis (in 3.5‐kDa cut‐off tubing) against H_2_O at 4°C for 24 h, with multiple changes of H_2_O. The extracted enzyme was then freeze dried, weighed, and stored at −80°C.

The culture filtrate collected in the first step was also dialysed and freeze dried as a source of soluble extracellular enzymes.

### Acyltransferase assays *in vivo* and *in vitro* with mono‐ and oligosaccharide acceptor substrates

Aliquots of 7‐day‐old spinach or Arabidopsis cell culture (10 μl; not washed) were incubated with ~200 Bq [^14^C]OxT (oxalyl donor substrate; to give a concentration of ~50 μm) and a potential acceptor substrate (such as a sugar; 5% w/v final concentration) for 0–16 h. Aliquots (10 μl) of cell‐free spent medium were similarly incubated with [^14^C]OxT and an acceptor substrate.

Alternatively, spinach and Arabidopsis enzyme preparations (see previous section) were assayed. The reaction mixture (final volume 10 μl) contained ~200 Bq [^14^C]OxT (making 50 μm), 5% w/v of a potential acceptor substrate such as a sugar, and 1% (w/v) enzyme preparation, in 10 mm PIPES [piperazine‐*N*,*N′*‐bis(2‐ethanesulfonic acid); Na^+^, pH 7].

After the incubation period, the reaction was stopped by addition of 0.1 volumes of formic acid. The whole sample (including any cells), after addition of 1 μl 10 mm Orange G as an internal marker, was loaded on to Whatman 3mm paper. HVPE (Fry, 2011) was conducted in pH 6.5 buffer (routinely containing 5 mm EDTA) at 2.5 kV for 30 min or in pH 2.0 buffer at 2.5 kV for 30 min. Products were detected by autoradiography, then quantified by scintillation counting in 2 mL ScintiSafe.

The acceptor substrates tested were commercial monosaccharides, sucrose, maltose, cellobiose and raffinose. We also tested a mixture of tamarind xyloglucan oligosaccharides (XGOs; predominantly a mixture of XLLG > XXLG > XXXG), kindly donated by Mr K. Yamatoya, Dainippon Pharmaceutical Co., Osaka, Japan.

### Acyltransferase assays *in vitro* with polysaccharide acceptor substrates

Polysaccharide‐impregnated paper was prepared by dipping Whatman 3mm paper slowly through a 1% solution of polysaccharide: methyl‐esterified pectin (from citrus fruit, >85% esterified), homogalacturonan (lacking methyl‐esters; from orange), xyloglucan (from tamarind) or xylan (from birch wood; heated to dissolve). The paper was then allowed to dry before being cut into 1.5 × 2.0‐cm rectangles.

Each small paper was rolled tightly, placed in a 0.2‐ml polymerase chain reaction (PCR) tube, and wetted with 35 μl of a solution containing 1% (w/v) enzyme, 10 mm PIPES (Na^+^, pH 7.0), and 0.67 μm of radiolabelled donor substrate (OxT or cOxT) or free OxA as a control. The tubes were sealed tightly and incubated for up to 24 h, then the paper was placed in 5 ml 70% ethanol or acidified ethanol (75% ethanol with 5% formic acid) and rotated on a wheel for 30 min. The ethanol was removed and a sample assayed for ^14^C by scintillation counting. The paper was then repeatedly washed in ethanol until no more radioactivity was released, and the washed paper was assayed for radioactivity by scintillation counting in the PPO/POPOP scintillant (which, unlike ScintiSafe, does not contain bases that might cleave ester bonds). Acyltransferase activity would result in radiolabelled paper, as the [^14^C]oxalate group from the donor substrate (OxT or cOxT) would have been linked to the ethanol‐insoluble polysaccharide–cellulose substrate by the formation of an ester bond.

## Conflict of interest

The authors declare no conflict of interest.

## Supporting information


**Figure S1.** [^14^C]Dehydroascorbic acid labels the polymeric fraction (AIR) of live Arabidopsis, rose and spinach cell cultures.
**Figure S2.** Effect of boiling or freeze–thawing on the ability of live spinach cells to transfer oxalate residues from oxalyl‐threonate into wall polymers.
**Figure S3.** Time‐course for transfer of oxalate residues from oxalyl‐threonate into wall polymers of live spinach cells.
**Figure S4.** pH dependence of long‐term *in‐vivo* spinach oxalyltransferase activity – transferring oxalate residues from oxalyl‐threonate to glucose.
**Figure S5.** pH dependence of short‐term *in‐vivo* spinach oxalyltransferase activity – transferring oxalate residues from oxalyl‐threonate to glucose.
**Figure S6.** Quantification of substrate and products of acyltransferase from Arabidopsis and spinach (time courses from Figure 5 in main manuscript).
**Figure S7.** *In‐vitro* oxalyltransferase activity (transferring oxalate residues from oxalyl‐threonate to glucose) increases with glucose concentration.
**Figure S8.** Inability of Arabidopsis oxalyltransferase to use amines as acceptor substrates.

## References

[tpj13984-bib-0001] Bartley, L.E. , Peck, M.L. , Kim, S.‐R. ***et al*** (2013) Overexpression of a BAHD acyltransferase, OsAt10, alters rice cell wall hydroxycinnamic acid content and saccharification. Plant Physiol. 161, 1615–1633. 23391577 10.1104/pp.112.208694 PMC3613443

[tpj13984-bib-0002] Bashline, L. , Lei, L. , Li, S. and Gu, Y. (2014) Cell wall, cytoskeleton, and cell expansion in higher plants. Mol. Plant, 7, 586–600. 24557922 10.1093/mp/ssu018

[tpj13984-bib-0003] Beisson, F. , Li, Y. , Bonaventure, G. , Pollard, M. and Ohlrogge, J.B. (2007) The acyltransferase GPAT5 is required for the synthesis of suberin in seed coat and root of Arabidopsis. Plant Cell, 19, 351–368. 17259262 10.1105/tpc.106.048033 PMC1820950

[tpj13984-bib-0004] Bellincampi, D. , Cervone, F. and Lionetti, V. (2014) Plant cell wall dynamics and wall‐related susceptibility in plant‐pathogen interactions. Front. Plant Sci. 5, 228. 24904623 10.3389/fpls.2014.00228 PMC4036129

[tpj13984-bib-0005] Bojarová, P. and Křen, V. (2009) Glycosidases: a key to tailored carbohydrates. Trends Biotechnol. 27, 199–209. 19250692 10.1016/j.tibtech.2008.12.003

[tpj13984-bib-0006] Brady, J.D. and Fry, S.C. (1997) Formation of di‐isodityrosine and loss of isodityrosine in the cell walls of tomato cell‐suspension cultures treated with fungal elicitors or H_2_O_2_ . Plant Physiol. 115, 87–92. 12223793 10.1104/pp.115.1.87 PMC158463

[tpj13984-bib-0007] Braidwood, L. , Breuer, C. and Sugimoto, K. (2014) My body is a cage: mechanisms and modulation of plant cell growth. New Phytol. 201, 388–402. 24033322 10.1111/nph.12473

[tpj13984-bib-0008] Brás, N.F. , Fernandes, P.A. and Ramos, M.J. (2010) QM/MM studies on the β‐galactosidase catalytic mechanism: hydrolysis and transglycosylation reactions. J. Chem. Theory Comput. 6, 421–433. 26617299 10.1021/ct900530f

[tpj13984-bib-0009] Brown, J.A. and Fry, S.C. (1993) Novel *O*‐d‐galacturonoyl esters in the pectic polysaccharides of suspension‐cultured plant cells. Plant Physiol. 103, 993–999. 8022945 10.1104/pp.103.3.993 PMC159074

[tpj13984-bib-0010] Burr, S.J. and Fry, S.C. (2009) Feruloylated arabinoxylans are oxidatively cross‐linked by extracellular maize peroxidase but not by horseradish peroxidase. Mol. Plant, 2, 883–892. 19825665 10.1093/mp/ssp044

[tpj13984-bib-0011] Carpita, N.C. and Gibeaut, D.M. (1993) Structural models of primary cell walls in flowering plants: consistency of molecular structure with the physical properties of the walls during growth. Plant J. 3, 1–30. 8401598 10.1111/j.1365-313x.1993.tb00007.x

[tpj13984-bib-0012] Cheynier, V. , Comte, G. , Davies, K.M. , Lattanzio, V. and Martens, S. (2013) Plant phenolics: recent advances on their biosynthesis, genetics, and ecophysiology. Plant Physiol. Biochem. 72, 1–20. 23774057 10.1016/j.plaphy.2013.05.009

[tpj13984-bib-0013] Chormova, D. , Messenger, D.J. and Fry, S.C. (2014a) Boron bridging of rhamnogalacturonan‐II, monitored by gel electrophoresis, occurs during polysaccharide synthesis and secretion but not post‐secretion. Plant J. 77, 534–546. 24320597 10.1111/tpj.12403 PMC4171739

[tpj13984-bib-0014] Chormova, D. , Messenger, D.J. and Fry, S.C. (2014b) Rhamnogalacturonan‐II cross‐linking of plant pectins via boron bridges occurs during polysaccharide synthesis and/or secretion. Plant Signal. Behav. 9, e28169. 24603547 10.4161/psb.28169 PMC4091542

[tpj13984-bib-0015] Chortyk, O.T. , Kays, S.J. and Teng, Q. (1997) Characterization of insecticidal sugar esters of *Petunia* . J. Agric. Food Chem. 45, 270–275.

[tpj13984-bib-0016] Collins, P.M. and Ferrier, R.J. (1995) Monosaccharides: their chemistry and their roles in natural products. Chichester, UK: Wiley & Sons.

[tpj13984-bib-0017] Cosgrove, D.J. (2005) Growth of the plant cell wall. Nat. Rev. Mol. Cell Biol. 6, 850–861. 16261190 10.1038/nrm1746

[tpj13984-bib-0018] Dalton, C.C. and Street, H.E. (1976) The role of the gas phase in the greening and growth of illuminated cell suspension cultures of spinach (*Spinacia oleracea*, L.). In Vitro, 12, 485–494. 965016 10.1007/BF02796491

[tpj13984-bib-0019] D'Auria, J.C. (2006) Acyltransferases in plants: a good time to be BAHD. Curr. Opin. Plant Biol. 9, 331–340. 16616872 10.1016/j.pbi.2006.03.016

[tpj13984-bib-0020] Dewhirst, R.A. , Clarkson, G.J.J. , Rothwell, S.D. and Fry, S.C. (2017) Novel insights into ascorbate retention and degradation during the washing and post‐harvest storage of spinach and other salad leaves. Food Chem. 233, 237–246. 28530571 10.1016/j.foodchem.2017.04.082 PMC5441274

[tpj13984-bib-0021] Dudareva, N. , D'Auria, J.C. , Nam, K.H. , Raguso, R.A. and Pichersky, E. (1998) Acetyl‐CoA:benzylalcohol acetyltransferase–an enzyme involved in floral scent production in *Clarkia breweri* . Plant J. 14, 297–304. 9628024 10.1046/j.1365-313x.1998.00121.x

[tpj13984-bib-0022] Esaka, M. , Imagi, J. , Suzuki, K. and Kubota, K. (1988) Formation of ascorbate oxidase in cultured pumpkin cells. Plant Cell Physiol. 29, 231–235.

[tpj13984-bib-0023] Eshdat, Y. and Mirelman, D. (1972) An improved method for the recovery of compounds from paper chromatograms. J. Chromatogr. A, 65, 458–459.

[tpj13984-bib-0024] Fleischer, A. , O'Neill, M.A. and Ehwald, R. (1999) The pore size of non‐graminaceous plant cell walls is rapidly decreased by borate ester cross‐linking of the pectic polysaccharide rhamnogalacturonan II. Plant Physiol. 121, 829–838. 10557231 10.1104/pp.121.3.829 PMC59445

[tpj13984-bib-0025] Foyer, C.H. and Halliwell, B. (1977) Purification and properties of dehydroascorbate reductase from spinach leaves. Phytochemistry, 16, 1347–1350.

[tpj13984-bib-0026] Foyer, C.H. and Mullineaux, P.M. (1998) The presence of dehydroascorbate and dehydroascorbate reductase in plant tissues. FEBS Lett. 425, 528–529. 9563527 10.1016/s0014-5793(98)00281-6

[tpj13984-bib-0027] Franková, L. and Fry, S.C. (2011) Phylogenetic variation in glycosidases and glycanases acting on plant cell wall polysaccharides, and the detection of transglycosidase and trans‐β‐xylanase activities. Plant J. 67, 662–681. 21554451 10.1111/j.1365-313X.2011.04625.x

[tpj13984-bib-0028] Franková, L. and Fry, S.C. (2013) Biochemistry and physiological roles of enzymes that ‘cut and paste’ plant cell‐wall polysaccharides. J. Exp. Bot. 64, 3519–3550. 23956409 10.1093/jxb/ert201

[tpj13984-bib-0029] Fry, S.C. (1986) Cross‐linking of matrix polymers in the growing cell walls of angiosperms. Annu. Rev. Plant Physiol. 37, 165–186.

[tpj13984-bib-0030] Fry, S.C. (2011) High‐voltage paper electrophoresis (HVPE) of cell‐wall building blocks and their metabolic precursors In The Plant Cell Wall – Methods and Protocols. (Popper Z. A., ed). Totowa, NJ: Humana Press, pp. 55–80. 10.1007/978-1-61779-008-9_4 21222076

[tpj13984-bib-0031] Fry, S.C. (2017) Plant cell wall polymers In Biofuels and Bioenergy. (Love J., Bryant J. A. and Butler C., eds). Oxford: Wiley‐Blackwell, pp. 59–88.

[tpj13984-bib-0032] Fry, S.C. , Willis, S.C. and Paterson, A.E.J. (2000) Intraprotoplasmic and wall‐localised formation of arabinoxylan‐bound diferulates and larger ferulate coupling‐products in maize cell‐suspension cultures. Planta 211, 679–692. 11089681 10.1007/s004250000330

[tpj13984-bib-0033] Fujiwara, H. , Tanaka, Y. , Yonekura‐Sakakibara, K. , Fukuchi‐Mizutani, M. , Nakao, M. , Fukui, Y. , Yamaguchi, M. , Ashikari, T. and Kusumi, T. (1998) cDNA cloning, gene expression and subcellular localization of anthocyanin 5‐aromatic acyltransferase from *Gentiana triflora* . Plant J. 16, 421–431. 9881162 10.1046/j.1365-313x.1998.00312.x

[tpj13984-bib-0034] Gille, S. and Pauly, M. (2012) *O*‐Acetylation of plant cell wall polysaccharides. Front. Plant Sci. 3, 12. 22639638 10.3389/fpls.2012.00012 PMC3355586

[tpj13984-bib-0035] Green, M.A. and Fry, S.C. (2005a) Apoplastic degradation of ascorbate: novel enzymes and metabolites permeating the plant cell wall. Plant Biosyst. 139, 2–7.

[tpj13984-bib-0036] Green, M.A. and Fry, S.C. (2005b) Vitamin C degradation in plant cells via enzymatic hydrolysis of 4‐*O*‐oxalyl‐l‐threonate. Nature, 433, 83–87. 15608627 10.1038/nature03172

[tpj13984-bib-0037] Harris, P.J. and Trethewey, J.A.K. (2010) The distribution of ester‐linked ferulic acid in the cell walls of angiosperms. Phytochem. Rev. 9, 19–33.

[tpj13984-bib-0038] Hemsley, P.A. , Kemp, A.C. and Grierson, C.S. (2005) The TIP GROWTH DEFECTIVE1 S‐acyl transferase regulates plant cell growth in *Arabidopsis* . Plant Cell, 17, 2554–2563. 16100337 10.1105/tpc.105.031237 PMC1197434

[tpj13984-bib-0039] Horemans, N. , Szarka, A. , Bock, M.De , Raeymaekers, T. , Potters, G. , Levine, M. , Banhégyi, G. and Guisez, Y. (2008) Dehydroascorbate and glucose are taken up into *Arabidopsis thaliana* cell cultures by two distinct mechanisms. FEBS Lett. 582, 2714–2718. 18619442 10.1016/j.febslet.2008.07.001 PMC2751764

[tpj13984-bib-0040] Ishii, T. (1997) Structure and functions of feruloylated polysaccharides. Plant Sci. 127, 111–127.

[tpj13984-bib-0041] Kärkönen, A. , Dewhirst, R.A. , Mackay, C.L. and Fry, S.C. (2017) Metabolites of 2,3‐diketogulonate delay peroxidase action and induce non‐enzymic H_2_O_2_ generation: potential roles in the plant cell wall. Arch. Biochem. Biophys. 620, 12–22. 28315301 10.1016/j.abb.2017.03.006 PMC5398285

[tpj13984-bib-0101] Kato, N. and Esaka, M. (1999) Changes in ascorbate oxidase gene expression and ascorbate levels in cell division and cell elongation in tobacco cells. Physiol. Plant. 105, 321‐329.

[tpj13984-bib-0042] Kauss, H. , Swanson, A.L. and Hassid, W.Z. (1967) Biosynthesis of methyl ester groups of pectin by transmethylation from *S*‐adenosyl‐l‐methionine. Biochem. Biophys. Res. Commun. 26, 234–240. 6030269 10.1016/0006-291x(67)90240-9

[tpj13984-bib-0043] Li, R. , Xin, S. , Tao, C. , Jin, X. and Li, H. (2017) Cotton ascorbate oxidase promotes cell growth in cultured tobacco bright yellow‐2 cells through generation of apoplast oxidation. Int. J. Mol. Sci. 18(7), 1346. 10.3390/ijms18071346 PMC5535839 28644407

[tpj13984-bib-0044] Lin, L.S. and Varner, J.E. (1991) Expression of ascorbic acid oxidase in zucchini squash (*Cucurbita pepo* L.). Plant Physiol. 96, 159–165. 16668145 10.1104/pp.96.1.159 PMC1080727

[tpj13984-bib-0045] Liso, R. , Tullio, M.C.De , Ciraci, S. ***et al*** (2004) Localization of ascorbic acid, ascorbic acid oxidase, and glutathione in roots of *Cucurbita maxima* L. J. Exp. Bot. 55, 2589–2597. 15520029 10.1093/jxb/erh262

[tpj13984-bib-0046] Luo, J. , Nishiyama, Y. , Fuell, C. ***et al*** (2007) Convergent evolution in the BAHD family of acyl transferases: identification and characterization of anthocyanin acyl transferases from *Arabidopsis thaliana* . Plant J. 50, 678–695. 17425720 10.1111/j.1365-313X.2007.03079.x

[tpj13984-bib-0047] Marry, M. , Roberts, K. , Jopson, S.J. , Huxham, I.M. , Jarvis, M.C. , Corsar, J. , Robertson, E. and McCann, M.C. (2006) Cell–cell adhesion in fresh sugar‐beet root parenchyma requires both pectin esters and calcium cross‐links. Physiol. Plant. 126, 243–256.

[tpj13984-bib-0048] May, M.J. and Leaver, C.J. (1993) Oxidative stimulation of glutathione synthesis in *Arabidopsis thaliana* suspension cultures. Plant Physiol. 103, 621–627. 12231968 10.1104/pp.103.2.621 PMC159023

[tpj13984-bib-0049] Mertz, D. (1961) Distribution and cellular localisation of ascorbic acid oxidase in the maize root tip. Am. J. Bot. 48, 405–413.

[tpj13984-bib-0050] Mertz, D. (1964) Ascorbic acid oxidase in cell growth. Plant Physiol. 39, 398–401. 16655933 10.1104/pp.39.3.398 PMC550092

[tpj13984-bib-0051] Mitchell, R.A.C. , Dupree, P. and Shewry, P.R. (2007) A novel bioinformatics approach identifies candidate genes for the synthesis and feruloylation of arabinoxylan. Plant Physiol. 144, 43–53. 17351055 10.1104/pp.106.094995 PMC1913792

[tpj13984-bib-0052] Offord, R. (1966) Electrophoretic mobilities of peptides on paper and thei use in the determination of amide groups. Nature, 211, 591–593. 5968723 10.1038/211591a0

[tpj13984-bib-0053] O'Neill, M.A. , Ishii, T. , Albersheim, P. and Darvill, A.G. (2004) Rhamnogalacturonan II: Structure and function of a borate cross‐linked cell wall pectic polysaccharide. Annu. Rev. Plant Biol. 55, 109–139. 15377216 10.1146/annurev.arplant.55.031903.141750

[tpj13984-bib-0054] Otte, O. and Barz, W. (1996) The elicitor‐induced oxidative burst in cultured chickpea cells drives the rapid insolubilization of two cell wall structural proteins. Planta, 200, 238–246.

[tpj13984-bib-0055] Parsons, H.T. and Fry, S.C. (2012) Oxidation of dehydroascorbic acid and 2,3‐diketogulonate under plant apoplastic conditions. Phytochemistry, 75, 41–49. 22226246 10.1016/j.phytochem.2011.12.005

[tpj13984-bib-0056] Parsons, H.T. , Yasmin, T. and Fry, S.C. (2011) Alternative pathways of dehydroascorbic acid degradation in vitro and in plant cell cultures: novel insights into vitamin C catabolism. Biochem. J. 440, 375–383. 21846329 10.1042/BJ20110939

[tpj13984-bib-0057] Pérez, S. , Rodríguez‐Carvajal, M. and Doco, T. (2003) A complex plant cell wall polysaccharide: rhamnogalacturonan II. A structure in quest of a function. Biochimie, 85, 109–121. 12765781 10.1016/s0300-9084(03)00053-1

[tpj13984-bib-0058] Perrone, P. , Hewage, C.M. , Thomson, A.R. , Bailey, K. , Sadler, I.H. and Fry, S.C. (2002) Patterns of methyl and *O*‐acetyl esterification in spinach pectins: new complexity. Phytochemistry, 60, 67–77. 11985854 10.1016/s0031-9422(02)00039-0

[tpj13984-bib-0059] Pettolino, F.A. , Walsh, C. , Fincher, G.B. and Bacic, A. (2012) Determining the polysaccharide composition of plant cell walls. Nat. Protoc. 7, 1590–1607. 22864200 10.1038/nprot.2012.081

[tpj13984-bib-0060] Polle, A. , Wieser, G. and Havranek, W.M. (1995) Quantification of ozone influx and apoplastic ascorbate content in needles of Norway spruce trees (*Picea abies* L., Karst) at high altitude. Plant, Cell Environ. 18, 681–688.

[tpj13984-bib-0061] Popper, Z.A. and Fry, S.C. (2008) Xyloglucan−pectin linkages are formed intra‐protoplasmically, contribute to wall‐assembly, and remain stable in the cell wall. Planta, 227, 781–794. 17987313 10.1007/s00425-007-0656-2

[tpj13984-bib-0062] Qi, B. , Doughty, J. and Hooley, R. (2013) A golgi and tonoplast localized *S*‐acyl transferase is involved in cell expansion, cell division, vascular patterning and fertility in Arabidopsis. New Phytol. 200, 444–456. 23795888 10.1111/nph.12385 PMC3817529

[tpj13984-bib-0063] Reid, M.E. (1941) Relation of vitamin C to cell size in the growing region of the primary root of cowpea seedlings. Am. J. Bot. 28, 410–415.

[tpj13984-bib-0064] Scheller, H.V. and Ulvskov, P. (2010) Hemicelluloses. Annu. Rev. Plant Biol. 61, 263–289. 20192742 10.1146/annurev-arplant-042809-112315

[tpj13984-bib-0065] Schilmiller, A.L. , Charbonneau, A.L. and Last, R.L. (2012) Identification of a BAHD acetyltransferase that produces protective acyl sugars in tomato trichomes. Proc. Natl Acad. Sci. 109, 16377–16382. 22988115 10.1073/pnas.1207906109 PMC3479610

[tpj13984-bib-0066] Schmelter, T. , Wientjes, R. , Vreeker, R. and Klaffke, W. (2002) Enzymatic modifications of pectins and the effect on their rheological properties. Carbohydr. Polym. 47, 99–108.

[tpj13984-bib-0067] Selvendran, R.R. (1975) Analysis of cell wall material from plant tissues: extraction and purification. Phytochemistry, 14, 1011–1017.

[tpj13984-bib-0068] Smirnoff, N. , Wheeler, G.L. and Loewus, F.A. (2000) Ascorbic acid in plants: biosynthesis and function. Crit. Rev. Biochem. Mol. Biol. 354, 291–314. 10.1080/10409230008984166 11005203

[tpj13984-bib-0069] Souza, W.R. de , Martins, P.K. , Freeman, J. ***et al*** (2018) Suppression of a single BAHD gene in *Setaria viridis* causes large, stable decreases in cell wall feruloylation and increases biomass digestibility. New Phytol. 218, 81–93. 29315591 10.1111/nph.14970 PMC5873385

[tpj13984-bib-0070] St‐Pierre, B. , Laflamme, P. , Alarco, A.M. and Luca, V.De (1998) The terminal *O*‐acetyltransferase involved in vindoline biosynthesis defines a new class of proteins responsible for coenzyme A‐dependent acyl transfer. Plant J. 14, 703–713. 9681034 10.1046/j.1365-313x.1998.00174.x

[tpj13984-bib-0071] Takahama, U. (1993) Redox state of ascorbic acid in the apoplast of stems of *Kalanchoe daigremontiana* . Physiol. Plant. 89, 791–798.

[tpj13984-bib-0072] Thompson, J.E. and Fry, S.C. (2001) Restructuring of wall‐bound xyloglucan by transglycosylation in living plant cells. Plant J. 26, 23–34. 11359607 10.1046/j.1365-313x.2001.01005.x

[tpj13984-bib-0073] Truffault, V. , Fry, S.C. , Stevens, R.G. and Gautier, H. (2017) Ascorbate degradation in tomato leads to accumulation of oxalate, threonate and oxalyl threonate. Plant J. 89, 996–1008. 27888536 10.1111/tpj.13439

[tpj13984-bib-0074] Weinhold, A. and Baldwin, I.T. (2011) Trichome‐derived *O*‐acyl sugars are a first meal for caterpillars that tags them for predation. Proc. Natl Acad. Sci. 108, 7855–7859. 21518882 10.1073/pnas.1101306108 PMC3093468

[tpj13984-bib-0075] Xu, D. , Shi, J. , Rautengarten, C. ***et al*** (2017) *Defective Pollen Wall 2* (*DPW2*) encodes an acyltransferase required for rice pollen development. Plant Physiol. 173, 240–255. 27246096 10.1104/pp.16.00095 PMC5210703

[tpj13984-bib-0076] Yang, Q. , Reinhard, K. , Schiltz, E. and Matern, U. (1997) Characterization and heterologous expression of hydroxycinnamoyl/benzoyl‐CoA:anthranilate *N*‐hydroxycinnamoyl/benzoyltransferase from elicited cell cultures of carnation, Dianthus caryophyllus L. Plant Mol. Biol. 35, 777–789. 9426598 10.1023/a:1005878622437

[tpj13984-bib-0077] Yeats, T.H. , Martin, L.B.B. , Viart, H.M.‐F. ***et al*** (2012) The identification of cutin synthase: formation of the plant polyester cutin. Nat. Chem. Biol. 8, 609–611. 22610035 10.1038/nchembio.960 PMC3434877

[tpj13984-bib-0078] Zechmann, B. , Stumpe, M. and Mauch, F. (2011) Immunocytochemical determination of the subcellular distribution of ascorbate in plants. Planta, 233, 1–12. 20872269 10.1007/s00425-010-1275-x PMC3015205

[tpj13984-bib-0079] Zhou, L.‐Z. , Li, S. , Feng, Q.‐N. , Zhang, Y.‐L. , Zhao, X. , Zeng, Y.‐L. , Wang, H. , Jiang, L. and Zhang, Y. (2013) Protein S‐ACYL TRANSFERASE10 is critical for development and salt tolerance in *Arabidopsis* . Plant Cell, 25, 1093–1107. 23482856 10.1105/tpc.112.108829 PMC3634679

