# Supplementary material for: Oxalyltransferase, a plant cell‐wall acyltransferase activity, transfers oxalate groups from ascorbate metabolites to carbohydrates
Source: Plant J. 2018 Jul 26;95(4):743–57. doi: 10.1111/tpj.13984 (PMC6099474; doi:10.1111/tpj.13984)
Supplement: Supplementary file 2 [file TPJ-95-743-s002.docx]

**Supporting Information Legends**

**Fig. S1. [^14^C]Dehydroascorbic acid labels the polymeric fraction (AIR) of live *Arabidopsis*, rose and spinach cell cultures.**

Mini-cultures of rose, spinach and *Arabidopsis* cells (250 mg fresh weight of cells in 500 µl of spent medium, 1 week after sub-culturing) were supplied with [^14^C]DHA (~0.5 µM final concentration). After 6 h the medium was removed and the cells were washed sequentially in water, 70% ethanol and (three times) in acidified 75% ethanol. The resulting insoluble material (AIR; predominantly cell-wall material) was assayed for radioactivity by scintillation counting. Each value is an average of three replicate cultures ±SE.

**Fig. S2. Effect of boiling on the ability of live spinach cells to transfer oxalate residues from oxalyl threonate into wall polymers.**

Cultured spinach cells (250 mg; 1 week after sub-culturing) were either incubated at 100°C in the culture medium for 30 minutes then cooled (‘boiled’) or not treated (‘untreated’), and then incubated for 6 h with [^14^C]OxT (approximately 5 µM final concentration) in 500 µl of buffer [20 mM acetate (pyridine^+^, pH 4.5), for boiled ] or spent medium [untreated]. The cells were then washed sequentially in water, 70% ethanol and (three times) in acidified 75% ethanol. The radioactivity present in the AIR (polysaccharide fraction) was finally assayed by scintillation counting.

**Fig. S3. Time-course for transfer of oxalate residues from oxalyl threonate into wall polymers of live spinach cells.**

Replicate spinach cell-suspensions (from a 7-day culture; 250 mg cells resuspended in 500 µl of spent medium) were fed 0.2 kBq [^14^C]OxT (final concentration ~ 5 µM). At the time-points indicated, the medium was removed and the cells were washed in H_2_O, 70% ethanol and (3 times in) acidified 75% ethanol. The radioactivity present in the AIR was assayed by scintillation counting. Each point is an average of 3 individual cultures ±SE.

**Fig. S4 . pH dependence of long-term *in-vivo* spinach oxalyltransferase activity — transferring oxalate residues from oxalyl threonate to glucose.**

Spinach cells (1 week after sub-culturing) were removed from their culture medium and 10-mg aliquots were incubated in 20 µl of buffers of various pH values along with [^14^C]OxT (approximately 50 µM) and glucose (**Glc**; 5% w/v final concentration), or no glucose (**None**). The buffers used were (all at 10 mM, adjusted with NaOH): for pH 3 and pH 4, l-tartaric acid; for pH 5 and pH 6, phthalic acid; and for pH 7, PIPES. After 16 h incubation the media were analysed by HVPE at pH 6.5 and the paper was autoradiographed (**a**). The bands of [^14^C]OxG and [^14^C]OxA were cut out of the paper and quantified by scintillation counting: **b** shows the production of OxG with and without glucose added as an acceptor substrate, and **c** shows the yield of total products (OxG and OxA) in the samples which contained glucose.

**Fig S5. pH dependence of short-term *in-vivo* spinach oxalyltransferase activity — transferring oxalate residues from oxalyl threonate to glucose.**

Details as in Fig. S4 except that—

(i) the buffer for pH 6.0 and pH 6.5 was phthalate, and that for pH 7.0 and pH 7.5 was PIPES;

(ii) samples were incubated for only 15 min (**a**), 1 h (**b**), or 4 h (**c**).

**Fig. S6: Quantification of substrate and products of acyltransferase from *Arabidopsis* and spinach (time courses from Fig. 5 in main manuscript).**

Cell-wall enzymes were salt-extracted from live cell cultures of (a) *Arabidopsis* and (b) spinach at various ages after sub-culturing, then dialysed, freeze dried and redissolved at 1% (w/v) in 10 mM PIPES (Na^+^, pH 7.0). The enzyme preparations (10 µl) were incubated with 50 µM [^14^C]oxalyl-threonate with or without 5% (w/v) glucose for 4 h (+glc and –glc respectively). Products (OxA and OxG) and remaining substrate (OxT) were separated by electrophoresis at pH 6.5 and autoradiographed. The radiolabelled spots (see Fig. 5) were carefully cut out and quantified by scintillation counting.

Abbreviations: OxA, oxalate; OxG, oxalyl-glucose; OxT, oxalyl-threonate.

**Fig. S7. *In-vitro* oxalyltransferase activity (transferring oxalate residues from oxalyl threonate to glucose) increases with glucose concentration.**

Salt-soluble enzymes from *Arabidopsis* (10 mg/ml final concentration) were incubated with [^14^C]oxalyl threonate (OxT) (~50 µM) and various concentrations of glucose (0–50% w/v) in 10 mM PIPES buffer (Na^+^, pH 7.0) for 4 h. Enzyme-free controls containing 5% or 50% glucose were also incubated. The reaction-mixtures were then analysed by HVPE at pH 6.5 and the paper was autoradiographed (a). The bands of oxalyl glucose (OxG) and free oxalate (OxA) were carefully cut out of the paper and quantified by scintillation counting. The increasing oxalyl glucose (OxG) production with increasing glucose concentration is shown in b, and the relative production of OxA and OxG is shown in c.

**Fig. S8. Inability of *Arabidopsis* oxalyltransferase to use amines as acceptor substrates.**

Salt-soluble *Arabidopsis* enzymes (10 mg/ml final concentration) were incubated with [^14^C]oxalyl threonate (OxT) (~ 50 µM) and various potential acceptor substrates (1.5% w/v) in 10 mM PIPES buffer (Na^+^, pH 7.0) for 4 h. Potential acceptors tested were Lys (lysine), His (histidine), Spd (spermidine), Spn (spermine), Put (putrescine), Poly-Lys (poly-lysine), Poly-His (poly-histidine) and Glc (glucose). Controls lacking either an acceptor substrate (‘No acceptor’) or the enzyme extract (‘No enzyme’) were also included. The 4-h products were analysed by HVPE at pH 2.0, along with non-radiolabelled markers, which were stained with ninhydrin (for amines) or AgNO_3_ (for sugars; LB = lactobionic acid). Orange G was also included as a marker (indicated in pencil). The radiolabelled portion of the paper was autoradiographed. OxT exhibits a double band on electrophoresis at pH 2 because two interconvertible isomers (3- and 4-*O*-oxalyl threonate) are partially resolved. The anode and cathode ends are indicated (+ve and –ve).

On electrophoresis at pH 2, hypothetical [^14^C]oxalyl amides of Lys and His are predicted to have a net charge of roughly −0.5 (e.g. ε-oxalyl lysine) or −0.1 (e.g. α-oxalyl lysine) and thus to migrate slowly towards the anode; those of Spd, Spn and Put will have little or no net charge and thus show up close to the origin; and [^14^C]oxalyl amides of polylysine or polyhistidine would have a net positive charge and thus migrate towards the cathode. On this basis, no oxalyl amides are evidenced under conditions suitable for formation of an oxalyl glucose (OxG) ester.
